# Emotional experiences of Korean correctional officers in response to incarcerated persons’ self-harm: a qualitative study

**DOI:** 10.3389/fpsyg.2025.1525875

**Published:** 2025-06-13

**Authors:** Ji-Hye Lee, Hyung-Ran Park

**Affiliations:** ^1^Department of Nursing, Hyejeon College, Chungnam, Republic of Korea; ^2^College of Nursing, Research Institute of Nursing Science, Chungbuk National University, Cheongju, Republic of Korea

**Keywords:** correctional facilities, humans, self-injurious behaviour, prison, qualitative research

## Abstract

This study aimed to understand the experiences of correctional officers who deal closely with incarcerated persons who self-harm. Self-harm among incarcerated persons not only threatens the physical health of these individuals but also takes a toll on the mental health of correctional officers. A qualitative research method was utilised, with data collected through in-depth interviews of 15 participants from March 2022 to November 2022 and analysed utilising Colaizzi’s method. The results contained seven themes categorised into three theme clusters: ‘Suffering from psychological threat and pressure’, ‘Negative emotions encroaching into daily life’, and ‘Seeking stability by shifting perspective’. The findings underscore the need for trained professionals and programmes to effectively address the complex mental health challenges faced by correctional officers as they deal with incarcerated persons who self-harm.

## 1 Introduction

Correctional facilities are rehabilitation institutions designed to detain incarcerated persons and curb their criminal tendencies (Tharshini et al., 2021). Incarcerated persons typically exhibit antisocial behaviour, verbal aggression, and high levels of physical aggressiveness (Tharshini et al., 2021; Bodecka-Zych et al., 2022). They generally possess personality traits that involve sensitivity, instability, and susceptibility to overreaction and hyperactivity (Tharshini et al., 2021; Bodecka-Zych et al., 2022). In cases where these personality traits coexist with mental health issues, incarcerated persons are at a higher risk of experiencing critical physical health crises, such as self-harm and suicide (Ryland et al., 2020; Favril, 2021). Compared to suicide incidents, self-harm occurs at a considerably higher rate (Ministry of Justice Correctional Services, 2023; McTernan et al., 2023). Self-harm is defined as a behaviour that damages one’s own body without intention (Ryland et al., 2020). In South Korean correctional facilities, self-harm occurs at a rate over seven times higher than the number of suicide attempts and actual suicides (Ministry of Justice Correctional Services, 2023). The prevalence of self-harm among prisoners ranges from 31 to 184 per 1,000 individuals (McTernan et al., 2023), significantly higher than the 1% observed in the general population (Klonsky, 2011).

Exposure to incarcerated persons’ self-harm incidents often leads to intense stress for correctional officers, resulting in various mental health issues (Hewson et al., 2022; Fusco et al., 2021). In most cases, while correctional officers must immediately request assistance and respond to emergencies based on the severity of the self-harm, they cannot open cell doors until additional support arrives, leaving them directly exposed to incarcerated persons inflicting self-injuries (Yoon, 2023). Correctional officers may encounter physical threats while attempting to stop self-injurious behaviours and emotional violence by being exposed to the harsher injury being carried out (Hewson et al., 2022; Fusco et al., 2021; Yoon, 2023; Smith et al., 2019).

Correctional officers are tasked with incarcerated persons’ management, which exposes them to challenges significantly compounded by the administrative tasks required following incarcerated persons’ self-harm incidents, including repeated investigations by superiors, disciplinary measures, and potential transfers (Hewson et al., 2022; Yoon, 2023). Consequently, correctional officers may contemplate resigning, and experience severe psychological distress, often manifesting as acute panic attacks (Smith et al., 2019). Furthermore, correctional officers exposed to incarcerated persons’ self-harm exhibit a higher prevalence of post-traumatic stress disorder compared with the general population (Fusco et al., 2021; Ellison and Jaegers, 2022). Correctional officers repeatedly exposed to incarcerated persons’ self-harm express a need for mental health counselling and support from correctional facility nurses and other professionals (Smith et al., 2019; Goodwin et al., 2021). However, facility nurses are usually overwhelmed with incarcerated persons’ healthcare responsibilities, and due to staffing shortages, officers’ mental health needs are deprioritised (Isaac Caro, 2021). Notably, while the expanded human rights agenda has improved incarcerated persons’ care and treatment (Williams et al., 2022), the emotional well-being of the officers overseeing them remains largely overlooked. The emotional issues suffered, exacerbated by inadequate support, have contributed to a high incidence of suicide among correctional officers (Fusco et al., 2021; Yoon, 2023; Smith et al., 2019; Ellison and Jaegers, 2022). Although the Korean Correctional Bureau acknowledges the importance of correctional officers’ mental health (Ministry of Justice Correctional Services, 2023), there is a lack of proper understanding of their emotional experiences stemming from exposure to incarcerated persons’ self-harm incidents and a need to provide suitable concrete support.

Given the significant impact of incarcerated persons’ self-harm on correctional officers, it is essential to adopt a health professional approach that focuses on understanding the emotional experiences of correctional officers and addressing the challenges faced by these officers. Few studies in several countries have explored mental health (Fusco et al., 2021; Moghimi et al., 2022; Viotti, 2016; Coulling et al., 2024) or emotional experiences (Smith et al., 2019; Ramluggun, 2013; Marzano et al., 2015) of correctional officers dealing with incarcerated persons’ self-harm. Korean studies on correctional incidents, including incarcerated persons’ self-harm, have primarily focused on the security aspects of correctional facilities (Ju and Park, 2021). To our knowledge, no studies have explored the experience of correctional officers dealing with incarcerated persons’ self-harm, despite the importance of mental health issues such as job burnout (Choi et al., 2020) among correctional officers in Korea.

Given the current lack of research on correctional officers’ experiences in Korea, this study applied a qualitative approach to understanding the essence of correctional officers’ emotional experiences regarding incarcerated persons’ self-harm. The phenomenological method delves into human experience by understanding the nature, meanings, and essential structure of lived experiences (Patton, 2020). This study aimed to explore the emotional experiences of correctional officers in response to incarcerated persons’ self-harm using a phenomenological method. The research question was ‘What are the emotional experiences of correctional officers when confronted with incarcerated persons’ self-harm?’

## 2 Methods

### 2.1 Design

This qualitative study employed a phenomenological approach to investigate the emotional experiences of correctional officers in response to incarcerated persons’ self-harm. The study adhered to the Consolidated Criteria for Reporting Qualitative Research (COREQ) checklist (Tong et al., 2007).

### 2.2 Participants and setting

This study was conducted at two correctional facilities in Chungbuk province, South Korea. Participants were recruited from correctional officers who had encountered scenes of incarcerated persons’ self-harm. Purposive sampling was utilised to select participants who had experience in managing self-harm incarcerated persons and could articulate related phenomena effectively (Campbell et al., 2020). Correctional officers who expressed willingness to participate in the study after being referred to them by the prison medical staff were included in the study. The sampling ceased at 15 participants, reaching data saturation.

### 2.3 Data collection

Data were collected from 7 March to 14 November 2022 with approval from the institutional review board of the researcher’s university. Participants were recruited with the agreement of the prison governor and the cooperation of the medical staff in a correctional facility. Participants were informed of the purpose and procedure of the study via a written explanation sheet. Written informed consent was provided voluntarily via a signature. Face-to-face in-depth interviews were conducted in a private room by the first author, who has experience in conducting qualitative research and interviews. Each interview lasted between 40 and 100 min and was repeated until no new information emerged. All interviews were recorded with a digital voice recorder and transcribed by one research assistant. During the interviews, the researcher paid close attention to the participants’ verbal and nonverbal responses, carefully taking fieldnotes on expressions that vividly captured their emotional states. This was recorded in the transcript in parentheses. The first interview questions started as open-ended, broad questions to encourage free responses: ‘What experiences did you have when faced with an incarcerated person’s self-harm?’ Follow-up questions were posed based on participants’ responses. Table 1 shows the key interview questions. During the interview, the researcher attempted to suspend judgement and maintain neutrality. The participants’ general characteristics with respect to sex, age, years of work experience, and number of witnessed self-harm incidents were obtained through self-reported questionnaires.

**Table 1:** Examples of key interview questions.

What experiences did you have when faced with an incarcerated person’s self-harm?
What were your feelings about incarcerated persons’ self-harm incidents?
How did you experience incidents of incarcerated persons’ self-harm?
Could you share your impressions about incarcerated persons’ self-harm incidents?
What does incarcerate persons’ self-harm mean to you personally?
How did experiencing incarcerated persons’ self-harm incidents affect you emotionally?
What personal changes did you notice in yourself after experiencing incarcerated persons’ self-harm incidents?

### 2.4 Analysis

The data were analysed based on Colaizzi’s (1978) phenomenological method. The recorded interviews were transcribed verbatim. Guided by Colaizzi’s seven-step method, the analysis was performed in the following manner. First, the collected data were thoroughly examined through repeated readings to grasp the overall meaning without distorting the participants’ meaning. Second, each transcript was carefully analysed to identify and extract significant statements related to correctional officers’ experiences after witnessing incarcerated persons’ self-harm incidents. Third, the extracted significant statements were thoroughly reviewed. In this process, redundancies were eliminated, and meanings were formulated in a way that expressed the participants’ intended meaning in more general terms. Fourth, themes and theme clusters were derived from the formulated meanings, involving grouping statements and organising them into coherent themes and overarching theme clusters. The raw data and theme clusters were carefully reviewed to ensure the participants’ intended meaning was accurately represented. The researchers repeated this process of reflecting on and reorganising themes and theme clusters. In this step, 29 formulated meanings were categorised into 7 themes and 3 theme clusters. Fifth, the analysed data were comprehensively described under each theme, providing a broad explanation of correctional officers’ experiences from their exposure to incarcerated persons’ self-harm. Sixth, the essential structure of the correctional officers’ emotional experiences was described by explaining the themes and theme clusters and presenting participants’ original statements. Finally, the research findings, including themes, theme clusters, and quotations, were presented to two participants to verify whether they accurately reflected their experiences, to validate the research findings.

To ensure the rigour of the research findings, Sandelowski’s (1986) criteria in qualitative research were applied in the following manner. To establish credibility, all data were recorded and transcribed verbatim, and the original data were utilised for analysis. To enhance the transferability of the findings, data collection was conducted comprehensively, and the context was described in extensive detail. To ensure auditability, the interview schedule and situations were meticulously documented utilising field notes and memos. To establish confirmability, the interviewer maintained a neutral stance throughout the interview process. The two researchers thoroughly discussed and resolved any coding and structural discrepancies until a consensus was reached.

### 2.5 Ethical statement

The studies involving humans were approved by the Chungbuk National University Institutional Review Board (CBNU-202111-HRHR-0192). Participants were given verbal explanations and written information sheets about the purpose and procedures of the study, the right to withdraw at any time without any repercussions, guaranteed protection of personal information, and assurances of confidentiality and anonymity. All participants confirmed and signed the informed consent form voluntarily before participating in this study.

## 3 Results

The Participants included twelve males and three females, with ages ranging from 33 to 58 years (median age: 45.1 years). Participants witnessed between 1 and 20 self-harm incidents among incarcerated persons, with a median frequency of 5.6 incidents (Table 2). Analysis of the collected data yielded seven themes, categorised into three distinct clusters: suffering from psychological threat and pressure, negative emotions encroaching into daily life, and seeking stability by shifting perspective (Table 3).

**Table 2:** General characteristics of participants (*N* = 15).

Participant number	Sex (age)	Work experience	Number of self-harm incidents witnessed by the participant
1	Male (42)	15 years and 4 months	3 incidents
2	Male (39)	10 years and 11 months	5 incidents
3	Female (46)	21 years and 5 months	6 incidents
4	Male (38)	9 years	4 incidents
5	Male (58)	32 years and 6 months	10 incidents
6	Male (33)	8 years and 6 months	10 incidents
7	Male (46)	20 years	5 incidents
8	Female (41)	14 years and 9 months	1 incident
9	Male (36)	12 years and 4 months	5 incidents
10	Female (46)	19 years and 4 months	4 incidents
11	Male (49)	18 years and 4 months	3 incidents
12	Male (45)	8 years and 10 months	1 incident
13	Male (51)	22 years and 10 months	more than 20 incidents
14	Male (54)	33 years and 9 months	5 incidents
15	Male (53)	27 years	3 incidents

**Table 3:** Formulated meaning, themes, and theme clusters in this study.

Theme clusters	Themes	Formulated meanings
Suffering from psychological threats and pressure	Left with fear	Disconcerted by a sudden incident Overwhelmed by fear Scene etched into memory like a burn scar Imprinted memory surfaces vividly
	Overwhelmed by duty and responsibilities	Burdened by the weight of responsibility Intimidated by investigations and reprimands Wanting to avoid the situation Struggling to maintain a stoic exterior
Negative emotions encroaching into daily life	Negative emotions regarding settling into everyday life	Feeling powerless against unreasonable demands exploiting the situation Surge of various negative emotions Agonised by persistent depression Overwhelming emotional fluctuations Venting work-related emotions on family
	Choosing to engage in superficial relationships to protect emotions	Doubting others’ intentions in social relationships Intensified fatigue from relationships due to ongoing distress Maintaining a distance from others in relationships
	Disturbed balance of health	Mental exhaustion Worrying about mental health due to surging negative thoughts Developing sleep disorders Stress manifesting as physical symptoms Depleted energy due to persistent tension
Seeking stability by shifting perspective	Finding ways to gain psychological stability	Understanding and accepting the inmate’ situation Reflecting on self Personal efforts to escape negative emotions Gaining emotional stability from support provided by loved ones Relying on faith
	Seeking external help for recovery	Seeking organisational intervention to gain emotional stability Struggling to overcome biases against mental counselling Need for detailed policy support

### 3.1 Suffering from psychological threat and pressure

The participants experienced profound bewilderment and fear when they encountered incarcerated persons’ self-harm incidents. These scenes were vividly etched into their memories, leaving indelible impressions akin to burn scars.

#### 3.1.1 Left with fear

Most participants, despite their preparedness, were caught off guard by the unexpected nature of incarcerated persons’ self-harm. The sight of incarcerated persons’ unfocused eyes and distressed appearance during self-harm episodes, coupled with the resulting trauma and blood loss, instilled a deep-seated fear in the interviewed participants. Moreover, the potential threat posed by the tools utilised for self-harm further intensified their apprehension. As mentioned by Participant 7: ‘I try to maintain composure, even when I see blood on the floor… I feign nonchalance… Back in the office, my heart races and my face pales… As our team rushed into the room, saying “Here we go,” they suddenly lashed out… I was terrified… I knew I had to restrain the act, but I could not bring myself to enter the cell. I feared I might get stabbed’.

For the participants, incarcerated persons’ self-harm incidents were etched into their memories like vivid photographs, leaving an indelible mark akin to a burn scar. When observing unusual behaviour among incarcerated persons, they found themselves involuntarily recalling past incidents, becoming hypersensitive to the possibility of self-harm. The participants reported discomfort from intrusive memories of these episodes, even outside work hours, and described their attempts to forget as ‘futile’ due to repeated exposure. Participants 1 and 10, respectively, shared: ‘The memory persists vividly, resurfacing suddenly even during work hours. When an incarcerated person displays unusual excitement or behaves atypically, rambling incoherently, the unsettling thought “Could they be self-harming?” suddenly intrudes [into] my mind. This possibility makes me deeply uncomfortable as it triggers those haunting memories. These emotions resurface as raw as they were in the moment’; and ‘When exposure to such scenes becomes a daily routine, attempting to erase them from memory becomes futile. … Just imagine witnessing 10 or 20 scenes in a row. … The afterimage of these scenes lingers, much like a burn. … Even if you undergo surgery or plastic surgery to conceal it, such emotional scars do not disappear. Those marks last forever’.

#### 3.1.2 Overwhelmed by duty and responsibilities

The participants had to account for incarcerated persons’ self-harm incidents and, in severe cases, were involved in legal disputes. They felt burdened by the prospect of collective accountability extending to their superiors during administrative investigations. Criticism and reproach from colleagues eroded their confidence and led to psychological distress. Participants repeatedly reviewed their actions during self-harm incidents, fearing that they could not avoid administrative reprimands. Participants 2 and 3 stated, respectively: ‘The on-site workers and support team… night shift manager, emergency responders… If the response is not smooth, quite a few people become implicated other than the security chief and warden, who are involved anyways. Those on-site, especially the person in charge, feel the greatest burden’; and ‘Should I call for help? … We need support. … It is really humiliating. I tend to be intimidated when faced with criticism. I have done everything I could within my power, but did I overlook something? … I guess I fear being reproached’.

The participants monitored the moods of incarcerated persons with a history of self-harm vigilantly. Aware that incarcerated persons quickly discern officers’ emotional states, they constantly checked themselves to maintain a neutral demeanour, consciously regulating their words and actions. They strived to foster positive relationships with incarcerated persons to prevent self-injurious behaviour. However, this often led to feelings of shame, as they frequently had to acquiesce to unreasonable demands from incarcerated persons. As shared by a Participant 7: ‘The most serious damage was the feeling of profound shame… that guy is a heinous criminal who harmed many people, yet I must cater to him! I am hyper-aware of my tone and actions… I try to appear resolute – just feigning, you know… Here, I take on a completely different appearance from my true self… Even while interacting with other incarcerated persons, I pay attention to that guy… Working under such constant vigilance itself is a big stressor’.

### 3.2 Negative emotions encroaching into daily life

The participants experienced a range of negative emotions stemming from their exposure to incarcerated persons’ self-harm incidents, which significantly impacted their daily lives and social relationships. They developed a persistent sense of distrust and suspicion towards others, leading to strained interpersonal interactions and adverse effects on their health.

#### 3.2.1 Negative emotions settling into everyday life

The unresolved negative emotions caused participants to react with heightened sensitivity and volatility to minor actions by their family members. This led to family members becoming wary of their moods, while the participants experienced self-loathing and regret for misdirecting their anger towards their loved ones. In the participants’ words: ‘When I get home, I feel drained and irritable. I just want to lash out at someone, like I am just waiting for someone to provoke me. One day, my anger had reached its boiling point when I arrived home [after a night shift]. I looked at my child and said, “Eat your bread and go to the kindergarten.” However, my child refused. Consequently, I just threw the bread and handkerchief out… Such anger is still within me and surfaces now and then’. (Participant 3); ‘My children are often on the receiving end of my frustration. I find myself yelling at them over trivial matters. For no reason, I vent my anger on my children and wife. In those moments, I think, “Why am I behaving this way? This should not be happening.” However, the anger takes control. It needs an outlet… Suppressing it only leads to outbursts somewhere else… It is incredibly tough. I ask myself, “Is this my limit?”… The self-loathing is intense… Immediate remorse and deep regret follow’. (Participant 7).

#### 3.2.2 Choosing to engage in superficial relationships to protect emotions

Exposure to incarcerated persons’ self-harm incidents caused participants to distrust others, impacting their social relationships. They became increasingly skeptical of others’ actions and intentions, critically evaluating even friendly approaches. Forming new relationships felt burdensome, leading them to prefer maintaining connections only with those with whom they were already familiar. To protect their emotional well-being, they intentionally adopted guarded relationships, even at the cost of reduced social interactions. Participants 6 and 10 stated, respectively: ‘I find it harder to trust people now… Even when I meet new people, I am very doubtful of them… On the surface, they act friendly and polite, but I know those kinds of people act differently behind your back… I think, I should not be fooled by this… and do not get too close. It just gets troublesome… So, it is best to get along comfortably only with people I know and trust’; and ‘I have lost interest in interacting with people. It is exhausting… I have already expended so much energy witnessing disturbing events at work. So, essentially, I have no expectations from others… I am not curious at all to get to know anybody. I just want to rest. At home, I prefer to limit my interactions to my family’.

#### 3.2.3 Disturbed balance of health

The participants described their exposure to incarcerated persons’ self-harm incidents as a mentally draining experience, likening it to living through a horror movie. They felt that such experiences even made them doubt their belief in human dignity and the value of respecting others. As shared by Participant 10: ‘That scene, you know, it was utterly shocking… It was as if I was not watching a horror movie in a theater, but actually living it… After witnessing such an extreme horror, I feel mentally drained… I think, If I were in an extreme situation, I might end up like that myself. It makes me lose my belief in human dignity and respect for people altogether’.

These mentally exhausting experiences also took a toll on participants’ physical health. They reported a wide range of symptoms, including headaches, elevated blood pressure, tremors, excessive sweating, and sleep disturbances. Dealing with incarcerated persons’ self-harm incidents consumed significant energy, leaving participants feeling utterly drained. The constant need to remain vigilant about unpredictable self-harm attempts led to cumulative fatigue. Participants 3 and 8, respectively, elaborated: ‘This job does not energise others. On the contrary, these people deplete even the positive energy from you… They just suck all my energy, making me feel constantly drained… It is as if all the energy in my body has been sapped… [After an incident,] I know I have used up all my energy on just one person’; and ‘My headaches and nausea would not stop… So, I went to the hospital and found that my blood pressure was alarmingly high… It was shocking. I am only in my early 40s… I wondered if I should start taking blood pressure medication. The situation felt incredibly unfair… Admittedly, I am intimidated when incarcerated persons not only self-harm but also curse or bang on walls. My hands tremble, I break out in a sweat, and my heart races… These symptoms often persist for several days’.

### 3.3 Seeking stability by shifting perspective

The participants managed their emotions through their own means. They reported that support from their social circle and personal spiritual beliefs contributed to their emotional stability. While some sought external help for emotional care, others expressed negative stereotypes associated with actively seeking mental health support. Above all, participants recognised the need for policy-level interventions to systematically address emotional challenges beyond personal biases.

#### 3.3.1 Finding ways to gain psychological stability

Through observing incarcerated persons, participants gained a broader understanding of their role in dealing with incarcerated persons’ self-harm. They found emotional stability by communicating with loved ones, engaging in hobbies, and practising their religious beliefs. They regarded managing incarcerated persons’ self-harm as an integral part of their duty and described their role as a final bulwark against the incarcerated person’s self-injurious behaviour as if they were listening to the incarcerated person’s unspoken pleas. Participants 3 and 12, respectively shared: ‘If I were to describe our work in one word, it would be… something like the final bulwark… Self-harm involves inflicting injury on one’s own body, right? It is as if the person is saying, ‘Look at me. I am struggling’. It feels like I am there as the last line of defence. Even if this person pushes further, I am the one who must stop them… The most fulfilling aspect of my life is spending time with friends. I make a conscious effort to meet with them regularly. I believe the greatest support I have received has come from my friends.’; and ‘When faced with difficult problems, I do not try to solve them on my own. Instead, I pray and ask God for help. For instance, when I encountered an issue with an incarcerated person, I prayed for a solution. I have had numerous experiences where the problem was genuinely resolved after praying. Moreover, viewing the situation through the lens of faith, rather than my human, wounded perspective, helped me see the incarcerated person as another person just like myself. This perspective shift was of great help.

#### 3.3.2 Seeking external help for recovery

The participants recognised the limitations of relying solely on personal efforts to overcome emotional difficulties and acknowledged the need for professional intervention. However, they were hesitant to seek active mental healthcare due to negative stereotypes. They expressed a desire for an accessible support system without time or location constraints to aid in their recovery. Additionally, they emphasised the need for expanding existing programmes and establishing universally beneficial systems similar to routine health check-ups. For instance, Participants 3 and 7, respectively, shared: ‘It would be beneficial if mental healthcare were universally accessible, without prioritisation. I hope they develop a discreet system that does not make people worry about being observed… Like health check-ups, it should be mandatory for everyone, with potential consequences for non-compliance. This might encourage even reserved individuals to participate. If people experience positive results after trying it, they may be more likely to seek it out the next time’; and ‘I am not sure whether it was last year or the year before, but I considered seeking psychiatric treatment and medication, yet I could not go through with it… Well, there is still a stigma attached to it. Taking psychiatric medication feels like a big step… I ended up dealing with it alone. However, if there were a support system in the workplace, like a counselling centre, or a way to consult someone, that would be helpful. I hope such a system becomes available… For staff working with incarcerated persons, regular mental health check-ups seem to be necessary’.

## 4 Discussion

This study examined the experiences of 15 correctional officers who encountered incarcerated persons’ self-harm incidents. Participants reported suffering from psychological threats and pressure, and the encroachment of negative emotions into their daily lives. To combat the negative effects of witnessing these incidents, the participants shifted their perspectives from emotional obsessions to routine life and actively sought stability.

### 4.1 Suffering from psychological threat and pressure

Even though correctional officers may be prepared for unforeseen incidents, incarcerated persons’ self-harm typically occurs unexpectedly (Ryland et al., 2020; McTernan et al., 2023), easily leading to agitation and confusion. In emotionally charged situations, correctional officers may struggle to fully utilise their training and experience a loss of control (Marzano et al., 2015). This difficulty in maintaining control can elevate stress levels and induce tension, necessitating organisational measures to anticipate self-harm incidents among incarcerated persons and provide officers with robust protection.

Correctional officers are involuntarily exposed to incarcerated persons’ self-harm and face direct mental violence. Moreover, while subduing self-harming incarcerated persons, they are subjected to aggressive language and physical threats, experiencing fear of injury or death (Ellison and Jaegers, 2022). Such threatening incidents can lead to post-traumatic stress disorder (PTSD), anxiety, panic, and depression (Fusco et al., 2021). These experiences create a state of emotional sensitivity, with traumatic memories continually impacting their daily lives, as psychological scars that cause distress when re-experienced. The intimidation resulting from threats and violence, coupled with the loss of control, hinders active incarcerated persons’ management, which could result in further PTSD for correctional officers (Ramluggun, 2013; Marzano et al., 2015). Therefore, it is essential to adopt a preventive management approach before the onset of symptoms, as effective management of such threatening and stressful incidents can significantly affect recovery.

Correctional officers are tasked with protecting incarcerated persons’ lives (Viotti, 2016) and preventing and managing self-harm incidents (Marzano et al., 2015). Consequently, they can be held legally accountable for the outcomes of incarcerated persons’ self-harm episodes (Hewson et al., 2022). This burden of responsibility and potential for blame, exacerbated by a system that primarily focuses on the results of self-harm incidents rather than prevention efforts, may lead correctional officers to avoid direct involvement in such situations (Marzano et al., 2015). To minimise personal liability, correctional officers tend to prioritise administrative procedures, such as conducting interviews with incarcerated persons, over direct incarcerated persons’ management (Ramluggun, 2013). These circumstances underscore the need for institutional reforms to streamline administrative procedures and establish a more balanced approach to accountability regarding incarcerated persons’ self-harm (Yoon, 2023). Furthermore, implementing a peer support programme, through which colleagues who have faced similar challenges can offer emotional and psychological assistance (Smith et al., 2019), could effectively address the emotional toll on correctional officers.

### 4.2 Negative emotions encroaching into daily life

This study revealed that correctional officers faced emotional challenges, including feelings of shame and loss of control, when incarcerated persons attempted self-harm to achieve their goals, gain hegemony within the incarcerated person’s power structure, or secure privileges in their prison life. When incarcerated persons deliberately disregarded officers’ instructions, officers not only suffered from diminished confidence, feelings of helplessness, and depression (Hewson et al., 2022; Marzano et al., 2015) but also struggled with self-directed management in their work environment (Marzano et al., 2015). Repeated exposure to self-harm incidents and persistent negative emotional experiences can impair correctional officers’ ability to effectively utilise their skills and respond appropriately, potentially leading to maladaptive behaviours and mental health concerns (Smith et al., 2019; Marzano et al., 2015; Harvey, 2014). Moreover, the availability of resources within correctional facilities has been shown to positively correlate with the maintenance of correctional officers’ well-being (Harvey, 2014). Therefore, the findings of this study underscore the necessity of developing and implementing effective intervention programmes within correctional facilities to assist officers in recognising and overcoming their negative emotions.

The participants in this study reported expressing unresolved negative emotions from work by directing frustration and anger towards their family members. This finding aligns with previous research indicating that correctional officers who experience incarcerated persons’ violence tend to vent anger on their close family members (Fusco et al., 2021; Huang et al., 2024). This suggests that correctional officers struggle to maintain a healthy work-life balance (Huang et al., 2024), potentially also causing distress to their family members. Because support from significant others serves as an important coping resource for correctional officers’ stress management and mental health (Ellison and Jaegers, 2022), expanding counselling services to include correctional officers’ families may be effective. This highlights the need for developing comprehensive healing programmes that address correctional officers’ challenges and the environmental realities of correctional facilities while offering support to their families. Such programmes will strengthen fundamental support resources by fostering family understanding and empathy.

Moreover, the negative emotions experienced by participants had a detrimental effect on their interpersonal relationships, intensifying feelings of distrust and exhaustion. Consequently, they were selective about social interactions. Previous research has shown that negative emotions hinder the formation of new relationships, with traumatic events serving as significant barriers to establishing trust with others (Hewson et al., 2022; Lee, 2020). To address the mental health concerns of correctional officers facing such emotional challenges, it is crucial to implement comprehensive support programmes. These programmes should focus on anger management and social skills training to enhance communication skills and foster healthier interpersonal relationships, enhanced self-esteem, stress management, and coping strategies.

Frequent exposure to threatening incidents, such as self-harm, can lead to serious sleep disturbances among correctional officers and pose significant risks to their physical well-being (Martinez-Iñigo, 2021). There have been reports of correctional officers engaging in self-harm themselves after witnessing such behaviour among incarcerated persons (Hewson et al., 2022). Given these concerns, it is crucial to establish a comprehensive mental health support system within correctional facilities, specifically designed to address the psychological needs of correctional officers.

### 4.3 Seeking stability by shifting perspective

In the face of psychological threats and pressure, the participants actively sought to regain emotional stability, steering away from the clutches of negative emotions that threatened to encroach on their daily lives. They achieved this through a process of understanding and accepting the incarcerated person’s situations while recognising the limitations of their roles. Previous studies have similarly shown that correctional officers accepted their inability to completely prevent self-harm incidents among incarcerated persons through willpower alone (Marzano et al., 2015). Likewise, community nurses intervening in self-harm cases overcame challenges by acknowledging the boundaries of their responsibilities (Leddie et al., 2022). Correctional officers overcame their difficulties by developing personal strategies to fend off negative emotions, such as engaging in hobbies away from the work setting and communicating with close friends and colleagues (Smith et al., 2019; Leddie et al., 2022). Social support emerged as a crucial factor in helping correctional officers cope with the negative emotions stemming from their encounters with incarcerated persons’ self-harm incidents (Hewson et al., 2022; Harvey, 2014). Personal spiritual beliefs also served as an anchor for their attitudes and thoughts when dealing with self-harm incidents, enhancing their empathy and providing psychological stability.

While the participants considered seeking professional medical assistance for difficulties, their prejudices and stereotypes about mental healthcare acted as significant barriers to action. The social culture in Korea, characterised by hypersensitiveness to others’ perceptions and aversion to mental healthcare, contributed to their reluctance to utilise such services (Kim et al., 2018). Many officers expressed a desire for mental health counselling, but their engagement with healthcare activities remained low (Harvey, 2014). These findings underscore the necessity of creating an environment where mental health services can be accessed without fear of social stigma.

The participants emphasised the importance of accessible counselling programmes and support systems within correctional facilities that could be utilised flexibly, without time or location constraints, to address emotional challenges. Research has shown that nurse-led mind–body relaxation interventions implemented in correctional settings effectively reduce physical tension and anxiety while improving sleep quality (Pralong et al., 2020). Developing and implementing programmes that leverage internal resources, such as mental health nurses already working in correctional facilities, would be an efficient and effective approach to providing mental healthcare for correctional officers.

### 4.4 Study limitations

This study conducted an in-depth analysis of the experiences of only 15 correctional officers from two correctional facilities in South Korea, which may limit the generalisability of the results to other cultural settings. Nevertheless, the results can provide valuable insights for improving the mental health of correctional officers working in the unique environment of correctional facilities worldwide.

## 5 Conclusion

This study utilised Colaizzi’s (1978) descriptive phenomenological method to explore the experiences of correctional officers who witnessed self-harm incidents among incarcerated persons. Analysis revealed that these officers face significant psychological challenges and pressures stemming from such incidents, with negative emotions encroaching on their daily lives. Specifically, they experience fear, a burden of accountability, and adverse emotional reactions, which take a toll on their health and personal relationships. In response to these challenges, officers made efforts to understand incarcerated persons and engage in self-reflection. While they actively pursued recovery and sought to regain stability, they recognised their need for external support systems. To address these unmet needs, increased attention, and support for correctional officers’ well-being are necessary, along with the development and implementation of targeted nursing interventions and organisational support systems. By providing a deeper understanding of the experiences of correctional officers who have witnessed incarcerated persons’ self-harm incidents, this study offers practical insights for developing intervention policies and programmes that reflect the reality of correctional facilities.

## Data Availability

The raw data supporting the conclusions of this article will be made available by the authors, without undue reservation.
